# Ferroelectric Nanoparticles in Liquid Crystals: Recent Progress and Current Challenges

**DOI:** 10.3390/nano7110361

**Published:** 2017-11-01

**Authors:** Yuriy Garbovskiy, Anatoliy Glushchenko

**Affiliations:** UCCS Biofrontiers Center and Department of Physics, University of Colorado Colorado Springs, Colorado Springs, CO 80918, USA; aglushch@uccs.edu

**Keywords:** liquid crystals, nanomaterials, ferroelectric nanoparticles, spontaneous polarization, aggregation, nanocolloids, electro-optics, ions

## Abstract

The dispersion of ferroelectric nanomaterials in liquid crystals has recently emerged as a promising way for the design of advanced and tunable electro-optical materials. The goal of this paper is a broad overview of the current technology, basic physical properties, and applications of ferroelectric nanoparticle/liquid crystal colloids. By compiling a great variety of experimental data and discussing it in the framework of existing theoretical models, both scientific and technological challenges of this rapidly developing field of liquid crystal nanoscience are identified. They can be broadly categorized into the following groups: (i) the control of the size, shape, and the ferroelectricity of nanoparticles; (ii) the production of a stable and aggregate-free dispersion of relatively small (~10 nm) ferroelectric nanoparticles in liquid crystals; (iii) the selection of liquid crystal materials the most suitable for the dispersion of nanoparticles; (iv) the choice of appropriate experimental procedures and control measurements to characterize liquid crystals doped with ferroelectric nanoparticles; and (v) the development and/or modification of theoretical and computational models to account for the complexity of the system under study. Possible ways to overcome the identified challenges along with future research directions are also discussed.

## 1. Liquid Crystals and Nanoparticles: Introduction

Nanoparticles in liquid crystals remain a hot topic of modern soft condensed matter research. This statement becomes obvious considering hundreds of published papers reviewed in multiple publications [1,2,3,4,5]. The rise of nanotechnology in late 1990s revitalized the idea, expressed by F. Brochard and P. G. de Gennes back in 1970, to change the properties of liquid crystals by mixing them with sub-micrometer magnetic particles [6]. Since that time various types of nanomaterials mixed with liquid crystals were studied including magnetic [7,8], ferroelectric [8,9], dielectric [8], semiconductor [8,10,11], metal [8,12], polymer [8], and carbon-based (nanotubes, fullerenes, etc.) [8,13,14] nano-dopants (for more detail please also refer to a recently published collective monograph [1]).

The dispersion of nanoparticles in liquid crystals proved to be a very fertile concept leading to the variety of interesting effects and new multifunctional materials [1,2,3,4,5,6,7,8,9,10,11,12,13,14]. Given a tremendous amount of existing literature on nanomaterials in liquid crystals, we narrowed the scope of this paper by considering liquid crystals doped with ferroelectric nanoparticles, a research topic pioneered by Y. Reznikov to whom we dedicate this topical review.

## 2. Liquid Crystals Doped with Ferroelectric Nanoparticles: A Brief Historical Overview

The very first paper reporting systematic studies of nematic liquid crystals doped with ferroelectric nanoparticles was published back in 2003 [15]. The major idea of the paper [15] was to increase the sensitivity of liquid crystals to the electric field and their electro-optical performance through mixing them with ferroelectric nanomaterials. To reduce the aggregation of ferroelectric nanoparticles, (i) they were functionalized with oleic acid; and (ii) their volume concentration was relatively low (<1%).

Main features of the diluted suspension of ferroelectric nanoparticles in nematic liquid crystals reported in paper [15] include (1) nearly 2-fold enhanced dielectric anisotropy; (2) nearly 2-fold lowering of the threshold voltage; (3) linear electro-optical response, or, in other words, the sensitivity of the suspension to the sign of the applied electric field, a property intrinsic to ferroelectric liquid crystals rather than to nematics. Very strong electric field generated by a ferroelectric nanoparticle along with alignment of these nanoparticles in liquid crystals were considered a major physical reason leading to the aforementioned features (1)–(3).

**Figure 1:**
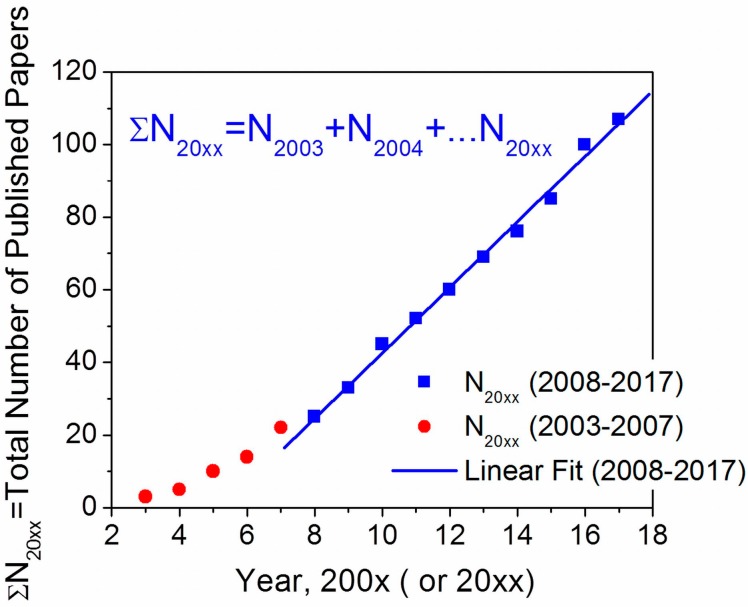
Total number of published papers reporting the properties of liquid crystals doped with ferroelectric nanomaterials versus time.

These findings, intriguing and very promising for applications, initiated very active research into the properties of liquid crystals doped with ferroelectric nanoparticles. Indeed, the total number of the published papers exhibits nearly linear increase during the last decade as shown in Figure 1. This figure also indicates high interest of the scientific community to this research topic.

**Figure 2:**
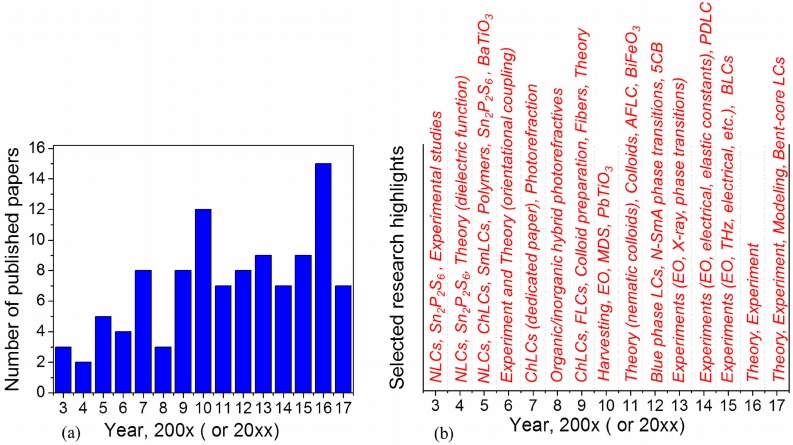
(**a**) Number of papers published during the 2003–2017 period; and (**b**) major research highlights. Nematic liquid crystals (NLCs), cholesteric liquid crystals (ChLCs), smectic liquid crystals (SmLCs), ferroelectric liquid crystals (FLCs), antiferroelectric liquid crystals (AFLC), polymer dispersed liquid crystals (PDLC), blue phase liquid crystals (BLCs), liquid crystals (LC), electro-optics (EO), molecular dynamics simulation (MDS).

A distribution of published journal papers along with major research highlights over the 2003–2017 periods are schematically shown in Figure 2 (published papers: 2003—[15,16,17]; 2004—[18,19]; 2005—[20,21,22,23,24]; 2006—[25,26,27,28]; 2007—[29,30,31,32,33,34,35,36]; 2008—[37,38,39]; 2009—[40,41,42,43,44,45,46,47]; 2010—[48,49,50,51,52,53,54,55,56,57,58,59]; 2011—[60,61,62,63,64,65,66]; 2012—[67,68,69,70,71,72,73,74]; 2013—[75,76,77,78,79,80,81,82,83]; 2014—[84,85,86,87,88,89,90]; 2015—[91,92,93,94,95,96,97,98,99]; 2016—[100,101,102,103,104,105,106,107,108,109,110,111,112,113,114]; 2017—[115,116,117,118,119,120,121,122,123]).

### 2.1. Early Developments (2003–2006)

During the first several years (2003–2006) practically all published papers [15,16,17,18,19,20,21,22,23,24,25,26,27,28] came from the same research team (Ukraine-US-UK). The materials of choice were ferroelectric nanoparticles (SPS = Sn_2_P_2_S_6_ and BTO = BaTiO_3_) dispersed in nematics [15,16,17,18,19,20,21,22,23,24,25,26,27,28], smectics [20,26,28], and cholesterics [20,26,28]. A simplified theory of ferroelectric nanoparticle/liquid crystal colloids was developed to explain experimental results [19,25,27].

### 2.2. Research Expansion (2007–2011)

Starting from 2007 more and more research groups became involved in studying the properties of liquid crystals doped with ferroelectric nanoparticles (Figure 2, [29,30,31,32,33,34,35,36,37,38,39,40,41,42,43,44,45,46,47,48,49,50,51,52,53,54,55,56,57,58,59,60,61,62,63,64,65,66]). At the same time, the scope of research interests significantly expanded. In addition to dielectric properties, electro-optics, and phase transitions thoroughly investigated during the 2003–2006 time period [15,16,17,18,19,20,21,22,23,24,25,26,27,28], photorefractive phenomena in organic–inorganic hybrids [32,33,35,37,38,46,50], polarization fluctuations observed in such systems [36], the effects of the nanoparticle size [56], and applications of liquid crystal/ferroelectric nanoparticle colloids for the design of electro-optical devices [31,39], tunable fibers [45] and alignment layers [44] were reported (see also Figure 2). The very first papers exclusively focused on ferroelectric smectic liquid crystals [41,55,66] and cholesteric liquid crystals [30,42] doped with ferroelectric nanoparticles also appeared.

Further progress in a theory of liquid crystals doped with ferroelectric nanoparticles was also made by considering how the orientational order of liquid crystals was affected by the orientational order of nanoparticles [47], by introducing Maier-Saupe-type theory of ferroelectric nanoparticles in nematic liquid crystals [65], and by analyzing Freedericksz transition in ferroelectric liquid-crystal nanosuspensions [64]. In addition, molecular dynamics simulations of spherical ferroelectric nanoparticles immersed in nematic liquid crystals were also performed [51].

Around the same time it became obvious that the properties of liquid crystals doped with ferroelectric nanoparticles depend strongly on the way ferroelectric nanoparticles are prepared [40,43,63,124]. As a result, the preparation of ferroelectric [40] and paraelectric [43] nanoparticles for their use in liquid crystal colloids and their applications was also thoroughly discussed. However, liquid crystals doped with ferroelectric nanoparticles turned out to be very delicate systems. As was shown in paper [48], even single component liquid crystals (5CB) doped with ferroelectric nanoparticles (SPS = Sn_2_P_2_S_6_) could exhibit different behavior such as increase and decrease in the threshold voltage and the “nematic-isotropic” phase transition temperature. Many factors could cause this very complex behavior [8,48,125]. For example, the electric field of nanoparticles could be screened by the charges in liquid crystals; nanoparticles could lose their ferroelectricity during their preparation; effective “dilution” of liquid crystals by nanoparticles, etc. [126]. These multiple factors could mask effects expected for liquid crystals doped with ferroelectric nanoparticles thus leading to the reported effects [8,125].

The ferroelectricity of nanoparticles was considered the major reason leading to the increased order parameter and the modification of the physical properties of the liquid crystal host [8,25,47,51,64,65]. That is why the need for the development of experimental methods allowing the production of truly ferroelectric nanoparticles suitable for their dispersion in liquid crystals became very urgent. An elegant method to harvest ferroelectric nanoparticles was reported in paper [49]. This technique was applied to study holographic beam coupling in inorganic-organic photorefractive hybrids using liquid crystals doped with harvested nanoparticles [50]. Moreover, the use of harvested ferroelectric nanoparticles revealed asymmetric Freedericksz transitions in symmetric liquid crystal cells doped with such harvested nanoparticles [57].

Since 2007, the choice of liquid crystals and ferroelectric nanoparticles was also gradually expanding by introducing new materials to study. For example, lead titanate (PTO) nanoparticles embedded in a liquid crystalline elastomer matrix and multiferroic BiFeO_3_ nanoparticles dispersed in partially fluorinated orthoconic antiferroelectric liquid crystal were studied in papers [54,66], respectively.

### 2.3. Research Expansion, Globalization, and Validation (2012–2017)

Research into the properties of liquid crystals doped with ferroelectric nanoparticles, initially undertaken by European and American scientists mostly, received a considerable boost due to the contributions coming from China, India, Iran, Taiwan, South Korea, and Japan. Several papers published in 2010–2011 [53,55,61,66] were followed up by an even greater number of publications [67,70,78,79,81,88,97,100,102,104,107,110,111,116,117,119]. For example, low voltage and hysteresis free blue phase liquid crystals doped with ferroelectric nanoparticles were reported [67,97]. Interesting effects of ferroelectric nanoparticles on the luminescence and electro-optics of ferroelectric liquid crystals were observed [78,81,85,89,90,95,96,105,106,107,108,109], and theory of (i) nanoparticles in ferroelectric liquid crystals [79]; (ii) the effect of ferroelectric nanoparticles on the isotropic-smectic-A phase transition [104] and the Freedericksz transition in smectic-A liquid crystals [119]; and (iii) the dielectric permittivity in the isotropic phase of the isotropic-smectic-A phase transition were also developed [117].

During the 2012–2017 time period the variety of materials used in experimental studies continued to grow. Polymer stabilized blue phase liquid crystals [97], polymer dispersed liquid crystals [88,110,111], and bent-core liquid crystals [122] doped with barium titanate (BTO) nanoparticles were studied. Moreover, lithium niobate (LNO) and multi-ferroic nanoparticles were introduced as ferroelectric dopants [85,99].

The effects of ferroelectric nanoparticles on the properties of a single component liquid crystal such as 5CB still remain among major research interest [72,107,108]. Electro-optics (including the Freedericksz transitions in nematic and smectic-A liquid crystals) [98,119]; dielectric [85,102,105,118,123], electrical [85,86,92,93,100,102,103,107,116,121,122], and viscoelastic [90,96,122] properties; phase transitions and pre-transitional effects [80,94,95,96,107,112,113,118,122,123] in liquid crystals doped with ferroelectric nanoparticles are also receiving due attention during this time period. Another new direction gaining interest of the scientific community includes studies of hybrid liquid crystal-ferroelectric nanoparticles composite materials in the terahertz and microwave regions [91,120].

A very important aspect of current research is an increasing use of the harvested nanoparticles in experimental studies with the goal to distinguish between direct effects of the nanoparticle’s ferroelectricity on the properties of liquid crystals and concurrent effects such as the dilution effect, screening effect, etc. [77,85,94,105,112,118,123].

## 3. Technology and Basic Properties of Liquid Crystals Doped with Ferroelectric Nanoparticles

### 3.1. Current Technology

Liquid crystals doped with ferroelectric nanoparticles are typically produced by mixing a small amount of nanomaterials with the liquid crystal host. Prior to making the liquid crystal nano-dispersion, nanoparticles can be either functionalized with the surfactant (in this case they are typically dispersed in an isotropic and non-ionic liquid carrier such as heptane or toluene) or they can be in the form of nano-powders (without any capping agent). The surfactant is needed to reduce the aggregation of nanoparticles. So far, oleic acid is the most widely used surfactant. However, this surfactant is not an ideal option since the affinity of its molecules to the surface of nanoparticles is rather moderate [125].

Once nanoparticles are dispersed in liquid crystals, the obtained dispersion is subject to sonication and/or prolonged shaking to mediate the tendency of nanoparticles to aggregate, and to allow the non-ionic liquid containing nanoparticles for its complete evaporation from the liquid crystal mixture.

**Figure 3:**
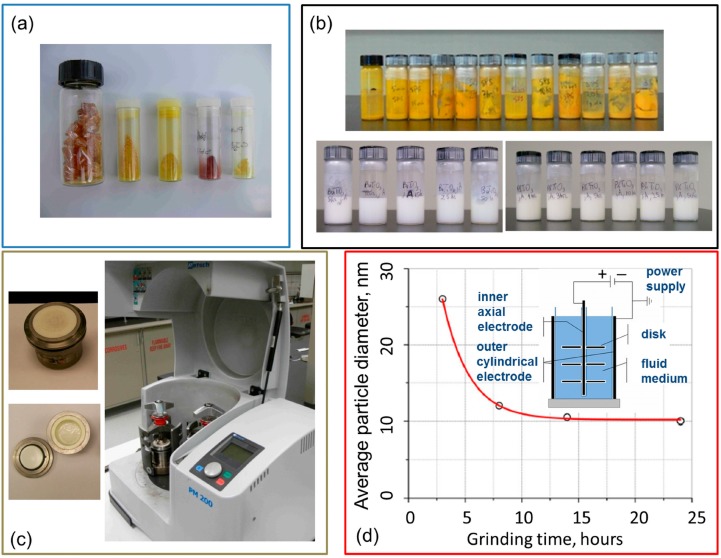
(**a**) Macro-crystals prior to milling; (**b**) The obtained dispersion of milled ferroelectric nanoparticles in a fluid carrier (heptane); (**c**) Commercially available high-energy ball mill; (**d**) an average size of the milled nanoparticles vs. grinding time [8]. A typical harvesting setup is shown in the inset (redrawn after [125]).

To date, the major method to produce ferroelectric nanoparticles for their applications in liquid crystal nano-colloids is a mechanical grinding of ferroelectric materials [8,40,125]. The ferroelectric material is milled together with the surfactant in a dielectric fluid carrier. The size of the obtained nanoparticles and their size distribution is governed by the milling time which strongly depends on the choice of materials and the design of the milling machine [8,40,125]. An example of the commercially available ball mill along with the “size vs. time” dependence is shown in Figure 3.

The “harvesting procedure” proposed in [49] is a very important step in the preparation of true ferroelectric nanoparticles by means of ball milling. The idea of this technique is to separate ferroelectric and non-ferroelectric nanoparticles by subjecting the dispersion of nanoparticles to a strongly non-homogeneous electric field. In this case nanoparticle with a non-zero permanent dipole moment will move whereas nanoparticles with net zero dipole moment will not “feel” the gradient of the electric field [49]. The schematic of the harvesting setup is shown in the inset, Figure 3d. A high DC (direct current) voltage (typically 10–20 kV) is applied across the inner wire electrode and the outer cylindrical electrode which is typically grounded. During the harvesting procedure, the harvested nanoparticles with permanent dipoles are accumulated on the inner wire electrode whereas non-ferroelectric nanoparticles remain in the fluid harvesting medium. Recent experiments with harvested ferroelectric nanoparticles provide new insights into the effects of their intrinsic ferroelectricity on the properties of liquid crystals [77,85,96,101,123]. It should be noted that ideal harvesting assumes non-charged ferroelectric nanoparticles. However, some ferroelectric nanomaterials such as SPS can be charged thus significantly complicating the harvesting process and interpretation of the obtained experimental results [92,125].

### 3.2. Basic Properties of Liquid Crystals Doped with Ferroelectric Nanoparticles

Consider ferroelectric nanoparticle immersed in a liquid crystal host. An electric field, $\vec{E}$, in the vicinity of ferroelectric nanoparticle approximated as a dielectric sphere with a spontaneous polarization, $\vec{P_{S}}$, can be estimated according to Equation (1) [125]:

(1) $$\vec{E}=\frac{R_{NP}^{3}}{3\varepsilon_{0}\varepsilon }\left(\frac{3\left(\vec{P_{S}}\cdot \vec{r}\right)\vec{r}}{r^{5}}-\frac{\vec{P_{S}}}{r^{3}}\right)$$

where $R_{NP}$ is a radius of nanoparticle, $\varepsilon_{0}$ is an electric constant, ε is a dielectric permittivity of the surrounding medium, and $\vec{r}$ is a radius vector. The value of this field is very high, on the order of 10^9^ V/m. This field can be comparable to or even stronger than the field due to intermolecular interactions in liquid crystals. Therefore, ferroelectric nanoparticles can directly affect the orientation of liquid crystal molecules in the vicinity of nanoparticles thus resulting in the field coupling between the liquid crystal director and the dipole moment of nanoparticles. In addition to the coupling with the liquid crystal director, this strong field can also change intermolecular interactions in the vicinity of nanoparticles [25,125]. These two factors can result in the increased ordering of liquid crystal molecules characterized by the order parameter S.

An increase in the order parameter changes basic properties of liquid crystals determined by S. The most important ones include the birefringence (Δn∝S), dielectric anisotropy (Δn∝S), and elastic constants (K∝S). Consequently, these changes should strongly affect the threshold voltage (the Freedericksz transition) of the liquid crystal nano-colloid [125]. In addition, an increased ordering of liquid crystals doped with ferroelectric nanoparticles can lead to the increase in the clearing temperature, $T_{c}$. More details on theory supporting these conclusions can be found in papers [25,47,65,84,87]. Here we just provide a brief summary of major theoretical results.

The effect of ferroelectric nanoparticles on the dielectric anisotropy and Freedericksz transition in nematic liquid crystals was analyzed in papers [19,27,64]. Assuming strong nanoparticle–liquid crystals coupling, the obtained mathematical expression for the threshold voltage was found to be similar to a standard expression for a pure nematic:

(2) $$V_{Fr}=\pi \sqrt{\frac{K}{\varepsilon_{0}\Delta \varepsilon_{eff}}}$$

where K is an elastic constant, $\Delta \varepsilon_{eff}$ is an anisotropy of the effective dielectric constant of the dispersion. Due to the spontaneous polarization of ferroelectric nanoparticles, the effective permittivity of liquid crystals/nanoparticles dispersion along the director is larger than the permittivity of pure liquid crystals. According to [64], a small volume fraction of ferroelectric nanoparticles (on the order of a few percent) is enough to reduce the threshold voltage by a factor of two.

An increase in the clearing temperature ($\Delta T_{c}$) of nematic liquid crystals doped with ferroelectric nanoparticles was analyzed in several papers [25,47,65]. Assuming perfect alignment of all ferroelectric nanoparticles along the liquid crystal director and applying the Maier-Saupe theory, the following expression was obtained [25]:

(3) $$\Delta T_{c}=\frac{zf_{v}{\left(\Delta \beta \right)}^{2}}{163.44\pi \varepsilon_{0}l_{m-m}^{3}k_{B}}P_{S}^{2}$$

where

z

is the nearest number of neighboring molecules separated by a distance $l_{m-m}$, $f_{v}$ is the volume fraction of nanoparticles, Δβ is the anisotropy of the molecular polarizability, $k_{B}=1.38\times 10^{-32}J/K$.

The consideration of the orientational distribution of nanoparticles in liquid crystals through the Landau theory along with the concept of coupled orientational order parameters for the liquid crystals and nanoparticles resulted in Formula (4) [47]:

(4) $$\Delta T_{c}=\frac{f_{v}\Delta \varepsilon }{135\rho_{LC}k_{B}\varepsilon_{0}\varepsilon^{2}}P_{S}^{2}$$

The generalization of the Landau theory [47] presented in paper [65] yielded another expression for the increase in the clearing temperature of nanocolloids:

(5) $$\Delta T_{c}=\frac{\pi f_{v}R_{NP}^{3}}{3T_{c}\rho_{LC}}{\left(\frac{2\Delta \varepsilon P_{S}^{2}}{675k_{B}\varepsilon_{0}\varepsilon^{2}}\right)}^{2}$$

In all cases (3)–(5) an increase in the clearing temperature is proportional to the concentration of nanoparticles. Both expressions (3) and (4) exhibit a quadratic dependence, $\Delta T_{c}\propto P_{S}^{2}$, whereas according to Formula (5) $\Delta T_{c}\propto P_{S}^{4}$. It should be noted that this scenario ($\Delta T_{c}\propto P_{S}^{4}$) was predicted assuming weak-interaction regime whereas expression (4) was obtained for the strong interaction regime (nanoparticles are small enough and the liquid crystal alignment is not distorted) [65].

## 4. Scientific and Technological Challenges

Early experimental and theoretical works, revealing an increased clearing temperature, enhanced order parameter, increased dielectric anisotropy and birefringence of liquid crystals doped with ferroelectric nanoparticles, stimulated very active research around the globe (Figure 1 and Figure 2). The reported findings were very promising from both academic and practical aspects. In fact, they showed a path toward a non-synthetic design of new liquid crystal materials by means of ferroelectric nanoparticles [8,20,21,26,31,125]. However, an expansion and globalization of the research into the properties of liquid crystals doped with ferroelectric nanoparticles identified many problems resulting in a poor reproducibility of the reported results. As an example, we compiled experimental data reported by independent research groups for the same single component liquid crystals (5CB) doped with ferroelectric nanoparticles (Table 1).

According to Table 1, even in the case of the same liquid crystals (5CB) the reported results [26,48,52,75,96,98,107,108] vary substantially. For example, there are papers reporting both increase [26,48,52] and decrease in the clearing point [48,96,107,108] of 5CB liquid crystals through doping them with ferroelectric nanoparticles. This variability of experimental data depends strongly on the material parameters of ferroelectric nanoparticles used in experiments. In some cases [75,107,108], the ferroelectricity of nanomaterials was not confirmed or checked. As a result, this type of experimental data is incomplete and prevents us from making any conclusions regarding possible effects of the nanoparticle ferroelectricity on the properties of liquid crystals. The size of nanoparticles also matters: the use of very large nanoparticles disturbs the director field [108]. Moreover, the prepared colloids are prone to aggregation and are very unstable. Aging phenomena and prehistory of the prepared ferroelectric nanomaterials are also important factors [48].

An analysis of Table 1 is very useful and instructive in identifying common challenges faced by scientists. These challenges can be broadly categorized into the following groups: (i) the control of the size, shape, and the ferroelectricity of nanoparticles; (ii) the production of a stable and aggregate-free dispersion of relatively small (~10 nm) ferroelectric nanoparticles in liquid crystals; (iii) the choice of ferroelectric nanomaterials and the selection of liquid crystals the most suitable for the dispersion of nanoparticles; (iv) the choice of appropriate experimental procedures and control measurements to characterize liquid crystals doped with ferroelectric nanoparticles; and (v) the development and/or modification of theoretical and computational models to account for the complexity of the system under study.

Let us discuss each of these groups in more detail.

### 4.1. Issues Related to Nanoparticles

Major issues associated with ferroelectric nanodopants include the control and evaluation of their size, shape, and the ferroelectricity.

As was already mentioned, the mechanical grinding of ferroelectric materials is a major technique to produce ferroelectric nanoparticles for their applications in liquid crystal colloids [8,40,125]. Using this method, the size of nanoparticles can be controlled by varying the milling time (Figure 3). The mechanical grinding is very delicate technique since the milling parameters should be optimized depending on the type of the mill and materials used (fluid carrier, surfactant, and ferroelectric). As a result, the produced ferroelectric nanoparticles should be carefully characterized prior to mixing them with liquid crystals.

Alternative methods to produce ferroelectric nanoparticles include physical (laser ablation, electrospinning); chemical (solid-state reaction; sol-gel technique; solvothermal method; hydrothermal method; molten salt method, and others); biological (biosynthesis) methods and their various combinations (please refer to a recent review [9] for more detail). These techniques could be very useful considering their potential to control both the size and shape of ferroelectric nanoparticles [9]. All these methods can be used in conjunction with the harvesting technique which is also very useful for the selection of truly ferroelectric nanoparticles.

To achieve good quality dispersion, relatively small ferroelectric nanoparticles are preferred. If the size of nanoparticle $R_{NP}$ is much less than the ratio $\frac{K}{W}$ (K is the elastic constant and W is the anchoring energy) then this nanoparticle does not disturb the liquid crystal director. Instead, the dispersed ferroelectric nanoparticles modify liquid crystals’ physical properties: temperature of liquid crystal phase transitions, birefringence, dielectric permittivity and magnetic permeability (if nano-multiferroics are used), conductivity, rotational viscosity, Freedericksz threshold, switching time etc. [125]. It should be noted that the size of ferroelectric nanomaterials can be reduced only to a certain critical value. Below this value ferroelectric materials typically lose their ferroelectric properties [101]. There is no universal agreement on the magnitude of this critical size since it depends on many factors including methods used to produce nanoparticles [101,127]. That is why ferroelectric properties of the newly produced nanoparticles should always be assessed by experimentalists.

Theoretical considerations described in previous section assume that nanoparticles are ferroelectric. If their ferroelectricity is not established in particular experiments, the obtained results cannot be used to verify existing theoretical predictions. Non-ferroelectric nanoparticles can affect the properties of the liquid crystal host through the dilution/anchoring effect [125,126]. For example, the change in the clearing temperature through the dilution/anchoring effect can be expressed as (6):

(6) $$\Delta T_{c}=-\left(1+B\right)f_{v}T_{c}^{0}$$

where B is the anchoring parameter, and $T_{c}^{0}$ is the clearing temperature of pure (non-doped) liquid crystals [126]. Pure dilution effect (B=0) can cause the reduction of the clearing temperature and the order parameter [126]. In the case of ferroelectric nanoparticles both competing effects (the effect of the nanoparticle’s polarization (Equations (3)–(5)) and the dilution/anchoring effects (Equation (6)) should be considered [112,118,123].

### 4.2. Stable and Aggregate-Free Dispersions

The production of a stable and aggregate-free dispersion of relatively small (~10 nm) ferroelectric nanoparticles in liquid crystals still remains a major challenge experimentalists are facing nowadays.

Strong Coulomb interactions between ferroelectric nanoparticles lead to their aggregation, and the presence of this aggregation was noted in many experimental works [8,125]. Even if the produced liquid crystal nano-colloids look stable and do not show any visible sign of aggregation, small aggregates can exist [8,125]. In this case the average size of aggregates is less than the wavelength of light. The aggregation reduces the effective spontaneous polarization of the colloid, change the effective concentration of nanoparticles, and alter the stability of the colloid making its macroscopic properties time dependent. As a result, experimental data and theoretical predications are not easy to compare.

To reduce the aggregation of ferroelectric nanoparticles, they are typically capped with a surfactant and their concentration in liquid crystals is kept at a relatively low level ($f_{v}<<10^{-2}$). Up to date, the oleic acid is the most widely used surfactant. As was already mentioned, this surfactant is not an optimized choice because of its low affinity to the surface of nanoparticles. As a result, some fraction of surfactants can be dissolved in liquid crystals causing the disordering of liquid crystals and contributing to the dilution effect. This effect can be strong enough to mask the effects due to ferroelectric nanoparticles. That is why it is a good laboratory practice to study possible effects of the used surfactant on the properties of liquid crystals.

Currently the search for better surfactants is underway [125]. Very promising are mesogenic surfactants recently applied to stabilize quantum dots and produce true dispersion of semiconductor nanoparticles in liquid crystals [128]. To achieve a stable dispersion of nanoparticles in liquid crystals, a mixture of an alkyl phosphonate and a dendritic surfactant containing promesogenic units was used [128]. This enabled the formation of thermodynamically stable colloids. The minimization of the distortion of the liquid crystal ordering around the nanoparticle was considered the major reason for the achieved stability (the shelf life of the prepared colloids was longer than 1 year).

### 4.3. Issues Associated with the Choice of Guest-Host Materials

So far, the suitability of particular ferroelectric nanomaterials for their dispersion in liquid crystals is practically not discussed in the literature. The majority of experimental studies were done using standard ferroelectric nanomaterials (SPS = Sn_2_P_2_S_6_, BTO = BaTiO_3_, LNO = LiNbO_3_) dispersed in nematic liquid crystals mostly (Figure 2). There are only a few publications reporting effects of multiferroic nanoparticles on the properties of liquid crystals [66,99]. Nematics are still the most widely used liquid crystal material (Figure 2). However, there is a tendency to broaden the number of liquid crystal materials by including cholesterics [30,42], different types of smectics [41,55,58,66,71,77,78,81,82,85,105,117,119], blue phase liquid crystals [67,97], polymer dispersed liquid crystals [88,110,111], and even bent-core liquid crystals [122]. One reason for this tendency is substantial improvements in the electro-optical performance of liquid crystal materials through doping them with ferroelectric nanoparticles. Faster electro-optical switching was reported for smectic liquid crystals doped with ferroelectric nanoparticles [77,78,85,105]. In the case of cholesteric liquid crystals, a minor quantity of ferroelectric nanoparticles can cause a 45% decrease of the driving voltage along with a two-fold increase in the effective dielectric anisotropy [42]. By mixing polymer-stabilized blue phase liquid crystals with ferroelectric nanoparticles, the vertical driving electric field was dramatically (~70%) reduced [97]. An enhancement of frequency modulation response time for polymer-dispersed liquid crystal doped with ferroelectric nanoparticles was also reported [110,111].

It should be noted that in practically all reported studies thermotropic liquid crystals are typically the material of choice. The use of lyotropic liquid crystals seems to be problematic since the highly polar molecules of water will inevitably screen the electric field of ferroelectric nanoparticles (for more detail regarding the electrical behavior of ferroelectric nanoparticles in aqueous medium we refer to [129]).

Multiphase thermotropic liquid crystals exhibit different types of mesophases which are typically achieved by changing the temperature of the sample. If this is the case, the temperature range of the mesophase should be below the Curie point of the ferroelectric nano-dopant. Ferroelectric nanoparticles lose their ferroelectricity above the Curie point. If the temperature of experimental samples is higher than the Curie point, the observed effects cannot be associated with the ferroelectricity of nanoparticles.

While selecting ferroelectric nanomaterials and liquid crystals for experimental studies, their possible contamination with ions should always be considered. Mobile ions always present in liquid crystals can screen (partially or even completely) the electric field of the ferroelectric nanoparticle. As a result, such “screened” ferroelectric nanoparticles will behave as nanodopants with significantly reduced (or even zero) effective permanent polarization. Consequently, an interpretation of the obtained experimental results and their comparison with theoretical predictions can become very problematic [125].

Strong Coulomb interactions between ions and ferroelectric nanoparticles dispersed in liquid crystals results in the well-known ion-trapping effect [2]. This effect, reported by many research groups, is very promising for the permanent purification of liquid crystals from ions. However, if ferroelectric nanoparticles are contaminated with ions prior to mixing them with liquid crystals, the observed effects can differ [130,131]. In fact, depending on the interplay between the adsorption-desorption processes in the liquid crystal nano-colloids and the level of the nanoparticle contamination, different regimes can be achieved: ion trapping regime [85,86,93], ion releasing regime [100], and no change in the concentration of ions [130]. Ferroelectric nanomaterials are very prone to uncontrolled ionic contamination. That is why it is important to assess the level of this contamination.

### 4.4. Experimental Procedures and Control Measurements

The choice of appropriate experimental procedures and control measurements to characterize liquid crystals doped with ferroelectric nanoparticles is not a trivial task. It can strongly affect the measured data. The electric field, originated from the spontaneous polarization of ferroelectric nanoparticles, and its interaction with surrounding mesogenic molecules is considered the major physical factor determining the properties of liquid crystal nano-colloids [125]. Therefore, an evaluation of this field through experimental assessment of the spontaneous polarization of nanoparticles is very important [63,125]. Electrical characterization of ferroelectric nanoparticles including measurements of the polarization switching current and ferroelectric hysteresis loop can provide enough information about the ferroelectricity of nanoparticles, possible screening effects, and the electric field due to the spontaneous polarization [63,124]. Complementary measurements such as Raman spectroscopy and X-ray structure analysis can identify the tetragonal structure of ferroelectric nanoparticles. However, these methods do not “see” the screening of the spontaneous polarization by mobile charges typically present in colloids.

Electrical and dielectrical measurements of liquid crystals doped with ferroelectric nanoparticles should be taken and interpreted with a high degree of caution. In the case of nematics, prior to applying the electric field, the net macroscopic polarization of the colloids is typically nearly zero (ferroelectric nematic suspensions exhibit behavior typical for paraelectrics). The applied electric field aligns dipoles of nanoparticles causing the amplification of the nanoparticle polarization. This amplification can affect the order parameter, phase transition temperatures, and the measured dielectric permittivity of the samples [112,118,123]. At the same time, techniques not utilizing electric field can yield different results for the same measured physical quantities. For example, the phase transition temperatures measured by means of dielectric spectroscopy were higher than that measured through differential scanning calorimetry [112,118]. Depending on its amplitude and frequency, the applied electric field can also alter the aggregation dynamics in liquid crystals doped with ferroelectric nanoparticles [60,63,124].

**Figure 4:**
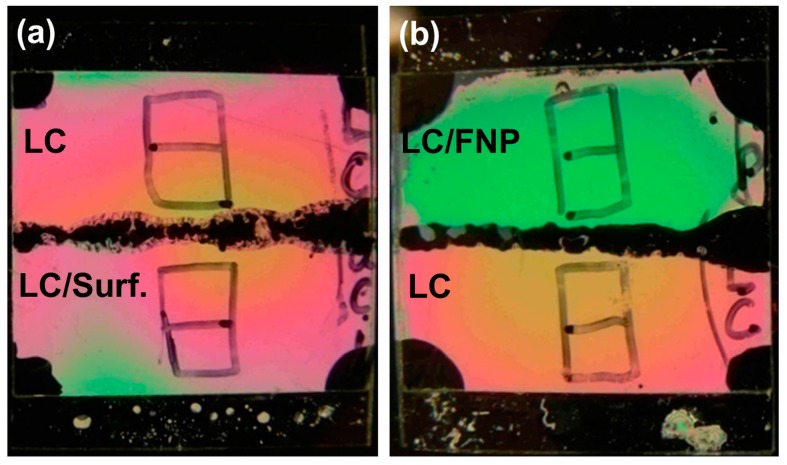
The twin cell placed in between two crossed polarizers: (**a**) the cell is filled with pure liquid crystals (this region is marked as “LC”) and liquid crystals doped with surfactant (oleic acid) (this region is marked as “LC/Surf.”); (**b**) the cell is filled with pure liquid crystals (marked as “LC”) and liquid crystals doped with ferroelectric nanoparticles (marked as “LC/FNP”).

The use of control samples in experiments is very important. Measurements performed with dispersions of nanoparticles in liquid crystals should be repeated with pure (non-doped) liquid crystals under identical conditions. In the case of optical and electro-optical studies, the use of the twin cells is very beneficial (Figure 4) [125]. The twin cell is the cell artificially divided by a polymer stripe into two identical regions. These regions are characterized by the same thickness, boundary conditions and anchoring strength. One of these regions is filled with liquid crystals doped with ferroelectric nanoparticles and the other region is filled with pure liquid crystals. Optical and electro-optical measurements performed with both regions can immediately reveal an impact of ferroelectric nanoparticles on the properties of liquid crystals (Figure 4).

### 4.5. New Theoretical and Computational Models

Existing theories [19,25,47,64,65,84,87,117,119] and computational models [51] considered rather simplified systems. Typically, the modeled system assumes mono-domain, neutral, and non-interacting ferroelectric nanoparticles dispersed in single component liquid crystals. At the same time, real systems are much more complex than the modeled ones. First of all, ferroelectric nanoparticles can vary in their size and the value of the spontaneous polarization. In addition, nanoparticles can aggregate and form dimers, chains, etc. In addition, nanoparticles are typically functionalized with the surfactant. Due to the finite affinity of the surfactant to the surface of nanoparticles, a fraction of this surfactant will also be dissolved in the liquid crystal host. The majority of liquid crystals are multi-component mixtures. As a result, there is a possibility for macro-nano separation of the liquid crystal components caused by ferroelectric nanoparticles. Another important aspect includes mobile charges in liquid crystals and the possibility of charged ferroelectric nanoparticles [92,125]. In addition, ferroelectric nanoparticles can be contaminated with ions prior to mixing them with liquid crystals [130,131]. All presented examples unambiguously illustrate how complex and delicate liquid crystals doped with ferroelectric nanoparticles are. More advanced theories and computational models should take into account the above-mentioned factors to ensure further progress in this very vibrant research.

## 5. Conclusions

Research into the properties of liquid crystals doped with ferroelectric nanoparticles has been carried out for more than a decade. Despite the broad variety of the results we can state that basic physics of *ferroelectric*, *non-charged and non-aggregated*, nanoparticles in *nematic* liquid crystals is reasonably understood. Ferroelectric nanoparticles do modify the properties of liquid crystals leading to the increase in the order parameter and resulting in improved electro-optical characteristics. This improved electro-optical performance (a higher birefringence, a shorter switching time, better contrast and lower threshold voltage, enhanced nonlinear-optical properties) is very promising for the design of the next generation devices which are cheaper, faster and better than currently existing products. These applications can include advanced displays, fast electro-optical switchers and shutters, memory cells, tunable filters, nonlinear-optical valves for optical processing systems, etc.

Additional studies are needed to explore a full potential of ferroelectric nano-dopants in cholesterics, various types of smectics, blue phase and bent core liquid crystals, and polymer dispersed liquid crystals. So far, among these materials, smectic liquid crystals are the mostly studied [41,55,58,66,71,77,78,81,82,85,105,117,119]. Only very limited number of papers considered cholesteric [30,42], blue phase [67,97], polymer dispersed [88,110,111] and bent core [122] liquid crystals doped with ferroelectric nanoparticles. Nevertheless, the available findings also indicate positive improvements in the properties of the aforementioned liquid crystals through doping them with ferroelectric nanoparticles.

Further progress in this vibrant research field will be achieved by introducing new experimental and theoretical concepts to solve major scientific and technological challenges discussed in the previous section. We hope our brief review will encourage students and professional researchers to “join the club” and actively participate in exploring new horizons of liquid crystals doped with ferroelectric nanoparticles, an exciting research direction launched by Prof. Y. Reznikov in early 2000s.

## Figures and Tables

**Table 1:** Single component nematic liquid crystals (5CB) doped with ferroelectric nanoparticles

Studied Samples	Observed Effects	Reference
Quasi-spherical (20 ± 10 nm) ferroelectric nanoparticles (Sn_2_P_2_S_6_, ~0.3 vol. %) were dispersed in 5CB. Nanoparticles were prepared by means of mechanical wet grinding. To provide the stability of nano-colloids, oleic acid was used as surfactant.	Several samples were prepared. The obtained results were dependent on the pre-history of the sample indicating possible aging of ferroelectric dispersions. As a result, both increase and decrease of the order parameter S (on the order of 5–10%) and of the clearing point $T_{c}$ (on the order of 1–10 deg.) was demonstrated.	[48]
Ferroelectric nanoparticles (BaTiO_3_; 1–4 vol. %; ~150 nm) were dispersed in 5CB. Nanoparticles were prepared by means of mechanical wet grinding. To provide the stability of nano-colloids, oleic acid was used as a surfactant.	An increase in the clearing point, $T_{NI}$, from 35.2 °C to 36.6 °C. The threshold voltage (the Freedericksz transition, $V_{Fr}$) was reduced from 0.79 V to 0.54 V. The order parameter was increased from 0.55 to 0.60. The turn-on time is decreased from 450 ms to 300 ms whereas the turn-off time is increased from 5.26 s to 7.75 s.	[52]
Ferroelectric nanoparticles (BaTiO_3_; ~1 wt. %; 30–50 nm) and (Sn_2_P_2_S_6_, ~200 nm) were dispersed in 5CB. Nanoparticles were prepared by means of mechanical wet grinding. To provide the stability of nano-colloids, oleic acid was used as a surfactant.	Signigicant (~2-fold) increase of the dielectric constants and 10–20% increase in the birefringence	[26]
Nanoparticles (BaTiO_3_; ~0.5 wt. %; ~4–40 nm) were dispersed in 5CB. Nanoparticles were prepared by means of mechanical wet grinding. The ferroelectricity of nanoparticles was not confirmed by experiments. To provide the stability of nano-colloids several surfactants including oleic acid were used.	An apparent shift of the Freedericksz transition towards a slightly higher value (according to electro-optical measurements) was not confirmed by capacitance measurements. The use of surfactants made of “nematogenic” molecules results in much more stable suspensions as compared to the use of oleic acid.	[75]
Ferroelectric nanoparticles (BaTiO_3_; 0.33–0.50 vol. %; ~50 nm) were dispersed in 5CB.	The threshold voltage (the Freedericksz transition, $V_{Fr}$) was reduced from 0.64 V to 0.56 V (0.33 vol. %) and 0.51 V (0.50 vol. %).	[98]
BaTiO_3_ nanoparticles (~100 nm; 0.05–5 wt. %) were dispersed in 5CB. No data on the ferroelectricity of the dispersed nanoparticles.	A decrease in the clearing point, $T_{c}$ (by about ~2 °C) was observed. The nematic temperature range is shortened with an increase in the concentration of nanoparticles. A decrease in the the dielectric anisotropy (from 13.1 to 11.2). The reduction of the threshold voltage from 1.02 V to 0.94 V. The splay elastic constant ($K_{11}$) is decreased from 16.50 pN to 11.13 pN (0.05 wt. %), 7.91 pN (0.5 wt. %), and to 8.88 pN (5.0 wt. %). Both increase (~100 times, at 0.05 wt. %) and decrease (~10 times, at 5.0 wt. %) in the electrical conductivity measured along the director was observed.	[107]
Ferroelectric nanoparticles (BaTiO_3_; ~0.2–0.4 wt. %; ~12 nm) were dispersed in 5CB. Nanoparticles were prepared by means of mechanical wet grinding and harvested. To provide the stability of nano-colloids (over a few months), oleic acid was used as a surfactant.	A decrease in the clearing point, $T_{c}$ (by about 2.5 °C) was observed. The enthalpy of this transition ($\Delta H_{NI}\approx 3\;J/g$) remains almost unchanged. The nematic temperature range is shortened with an increase in the concentration of nanoparticles. Practically no change in the birefringence and the dielectric anisotropy. The splay elastic constant ($K_{11}$) is practically not affected by nanoparticles while the bend elastic constant(*K*_33_) decreases (~20%). The decrease (~20%) in the rotational viscosity $\gamma_{1}$.	[96]
Relatively large BaTiO_3_ particles (~600 nm; ~1 wt. %) were dispersed in 5CB (oleic acid was used as a surfactant).	A decrease in the clearing point, $T_{NI}$, from 35.2 °C to 32.4 °C, and in the rotational viscosity $\gamma_{1}$ from 0.081 Pa·s (pristine liquid crystals) to 0.078 Pa·s (liquid crystal nanocolloids). The reduction of the Freedericksz transition from 1.3 V to 0.3 V. The switching time: the turn-on time is decreased (~10%) whereas the turn-off time is increased (>50%).	[108]
